# Community-based cluster randomized controlled trial: empowering households to identify and provide appropriate care for low-birthweight newborns in Nepal

**DOI:** 10.1186/s12889-020-09317-w

**Published:** 2020-08-24

**Authors:** Stephen Hodgins, Binamra Rajbhandari, Deepak Joshi, Bharat Ban, Subarna Khatry, Luke C. Mullany

**Affiliations:** 1 School of Public Health, University of Alberta, Edmonton Clinic Health Academy, 11405 – 87 Ave, Edmonton, Alberta T6G 1C9 Canada; 2 International Rescue Committee, 38 Main Motor Road, Wilberforce, Freetown, Sierra Leone; 3 Save the Children, Nepal Country Office, Airport Gate Area, Shambhu Marg, Kathmandu, Nepal; 4 Independent consultant, Kathmandu, Nepal; 5 Department of International Health, Johns Hopkins Bloomberg School of Public Health, Baltimore, MD 21228 USA

**Keywords:** Low birth-weight, Thermal care, Care-seeking, Anthropometry, Home childbirth, Behavior change, Sensitivity and specificity, Low- and middle-income countries

## Abstract

**Background:**

Most newborn deaths occur among those of low birthweight (LBWt), due to prematurity &/or impaired fetal growth. Simple practices can substantially mitigate this risk. In low-income country settings where many births occur at home, strategies are needed that empower mothers to determine if their babies are at higher risk and, if so, to take measures to reduce risk. Earlier studies suggest that foot-length may be a good proxy for birthweight. An earlier Nepal study found a 6.9 cm cut-off performed relatively well, differentiating normal from low birthweight.

**Methods:**

Community-based, cluster-randomized controlled trial. Objective: to determine whether family-administered screening, associated with targeted messages improves care practices known to mitigate LBWt-associated risks. Participants: women participating in a parent trial in rural Nepal, recruited late in pregnancy. Women were given a 6.9 cm card to assess whether the baby’s foot is small; if so, to call a number on the card for advice. Follow-up visits were made over the 2 weeks following the birth, assessing for 2 behavioral outcomes: reported skin-to-skin thermal care, and care-seeking outside the home; assessed restricting to low birthweight (using 2 cutoffs: 2500 g and 2000 g). Randomization: 17 clusters intervention, 17 control.

The study also documented performance along the presumed causal chain from intervention through behavioral impact.

**Results:**

2022 intervention, 2432 control. Intervention arm: 519 had birthweight < 2500 g (vs. 663 among controls), of which 503 were available for analysis (vs. 649 among controls). No significant difference found on care-seeking; for those < 2500 g RR 1.13 (95%CI: 0.97–1.131). A higher proportion of those in the intervention arm reported skin-to-skin thermal care than among controls; for those < 2500 g RR 2.50 (95%CI: 2.01–3.1). However, process measures suggest this apparent effect cannot be attributed to the intervention; the card performed poorly as a proxy for LBWt, misclassifying 84.5% of those < 2000 as normal weight.

**Conclusions:**

Although the trial found an apparent effect on one of the behavioral outcomes, this cannot be attributed to the intervention; most likely it was a result of pure chance. Other approaches are needed for identifying small, at-risk babies in such settings, and targeting them for appropriate care messaging.

**Trial registration:**

ClinicalTrials.gov NCT02802332, registered 6/16/2016.

## Background

Globally, it is estimated that approximately 15% of newborns weigh < 2500 g at birth; rates are the highest in South Asia, where low-birthweight newborns make up over a quarter of births [1]. In Nepal—the setting for the study reported in this paper—32% of newborns were found to weigh < 2500 g, in a rural terai (plains) population [2]. Most newborn deaths are among such babies of low birth-weight, either those growth-restricted in utero or born preterm; globally, although “low-birthweight babies constitute only about 14% of children born, they account for 60–80% of neonatal deaths” [3].

In principle, many deaths in small newborns could be prevented with good attention to thermal care [4] and breastfeeding [5] (early initiation, exclusive, at adequate frequency), and with prompt medical attention in case of complications. For institutional births, while mother and newborn are still in hospital there is an opportunity to identify such higher-risk newborns, provide them with any needed special care, and make suitable arrangements for follow-up after discharge (though frequently in low-resource settings these newborns do not receive such care). For home births, however, babies at high risk due to low birthweight are often not recognized as such and, therefore, may not receive needed care. In a Nepal setting, it has been found that mother’s judgement of relative size of their newborns is generally not reliable [6]. From the most recent Nepal Demographic and Health Survey [7], in rural areas fewer than half of births were in health facilities (44%) and among those in the bottom wealth quintile, only one third.

Nepal benefits from a comparatively robust peripheral-level primary healthcare system, consisting of health posts, serving populations of 5–10,000, staffed by 3 or more fulltime, paid health auxiliaries, who are supported by a network of 9 or more Female Community Health Volunteers (FCHV), most of whom are actively involved in program work—including maternal-child activities—notably advice and support to pregnant women. In a recent, nationally-representative survey of women who had given birth over the preceding year [8], 55% reported having received such support during their last pregnancy.

**Fig. 1:**
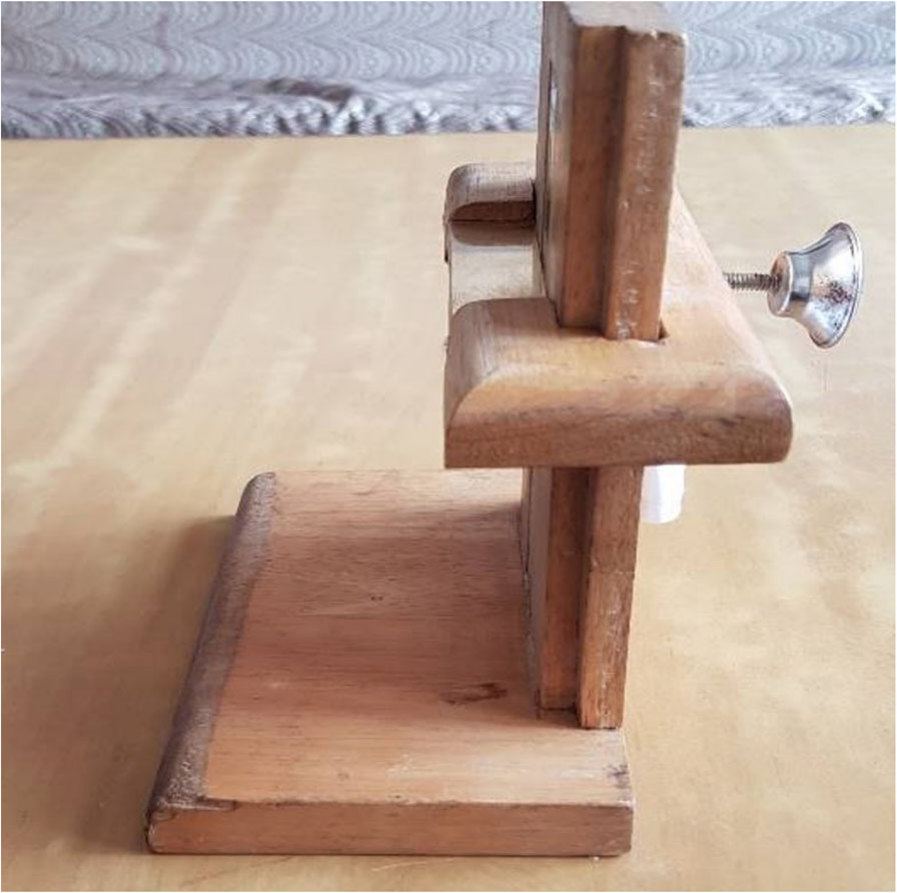
Device used in earlier study [2]

Based on the principle of the household production of health [9], we hypothesized that there may be ways to empower mothers and other household care providers—particularly in instances where births happen at home—to determine, themselves, if their babies are particularly small and, if so, to take appropriate actions to reduce risk. Earlier work in Nepal [2] documented good correlation between birthweight and several other anthropometric measures, notably foot-length. Using 6.9 cm as a cut-off, only 12.5% of those weighing < 2000 g would be falsely classified as normal weight and, of those > 2500 g, only 5.8% would be classified as small, potentially needing special care. A dedicated measuring box was used for that study (see Fig. 1).

We speculated that if pregnant women were given a card measuring 6.9 cm, with instructions on its use, they would be able to determine with reasonable accuracy whether their baby was at particular risk due to low birthweight. For such cases, we could target appropriate care messages.

Similar ideas have occurred to other investigators [10, 11], and a variety of strategies have been tested, most often involving issuing a ruler or other measuring device or materials to community health workers, who were then expected to make early postnatal home visits to identify small babies. Although the Ministry of Health and partners in Nepal have attempted a similar strategy (with the community health volunteers using Salter scales to identify small babies), this did not perform well (not only was coverage low but even when first piloted, many FCHVs had difficulty accurately weighing newborns) [12]. We were—instead—looking for an approach that would empower family members, themselves, to determine if their babies were particularly small.

**Fig. 2:**
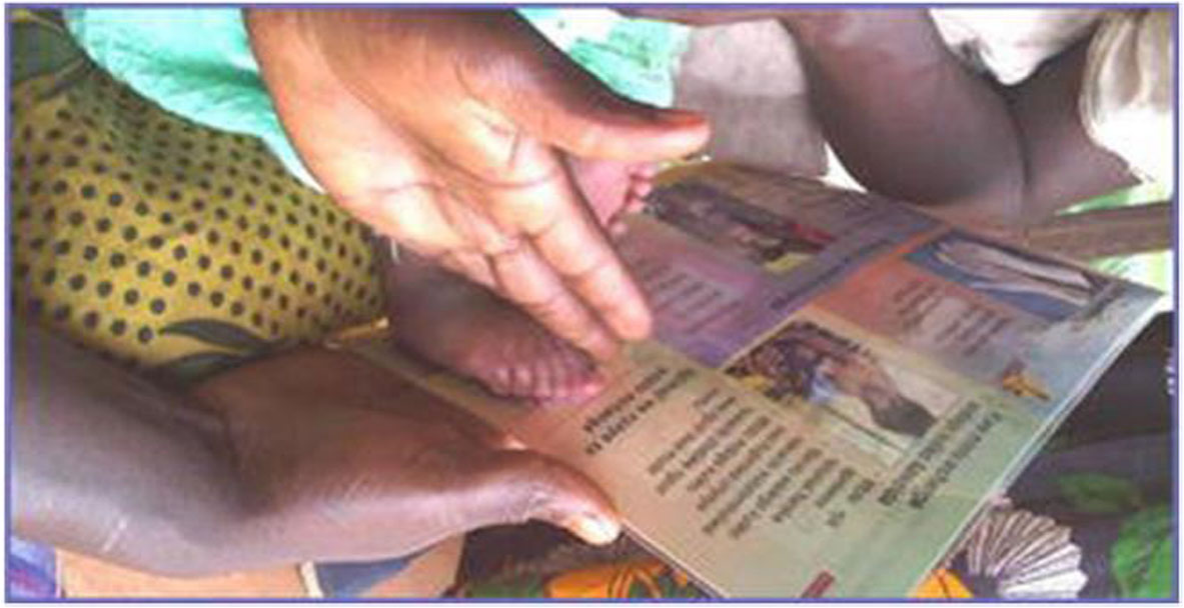
Counseling/ measurement card (from Marchant 2014 [10])

Experiences elsewhere are of some relevance. Marchant and colleagues [10] used a somewhat similar approach in a pilot in southern Tanzania. Community health workers (CHW) were given a laminated counseling card with an integrated measurement area on the bottom right corner of the card—as below (Fig. 2)—to classify newborns by foot-length as: very short (< 7.0 cm), short (7.0–7.9 cm), or not short (≥8.0 cm); and the CHWs were also instructed to measure and record foot-length, using a transparent plastic ruler.

In a Nepal-based study in a tertiary-level hospital [13], study staff did measures of newborn foot-length using 3 different methods, including use of rulers and tape-measures. There was some variation in results between the 3 methods, with average length varying by 0.2 cm between the most disparate. With the best performing method (using a transparent ruler), for a birthweight cutoff of 2000 g, a 7.5 cm threshold performed best, with sensitivity of 83% and specificity of 85%.

**Fig. 3:**
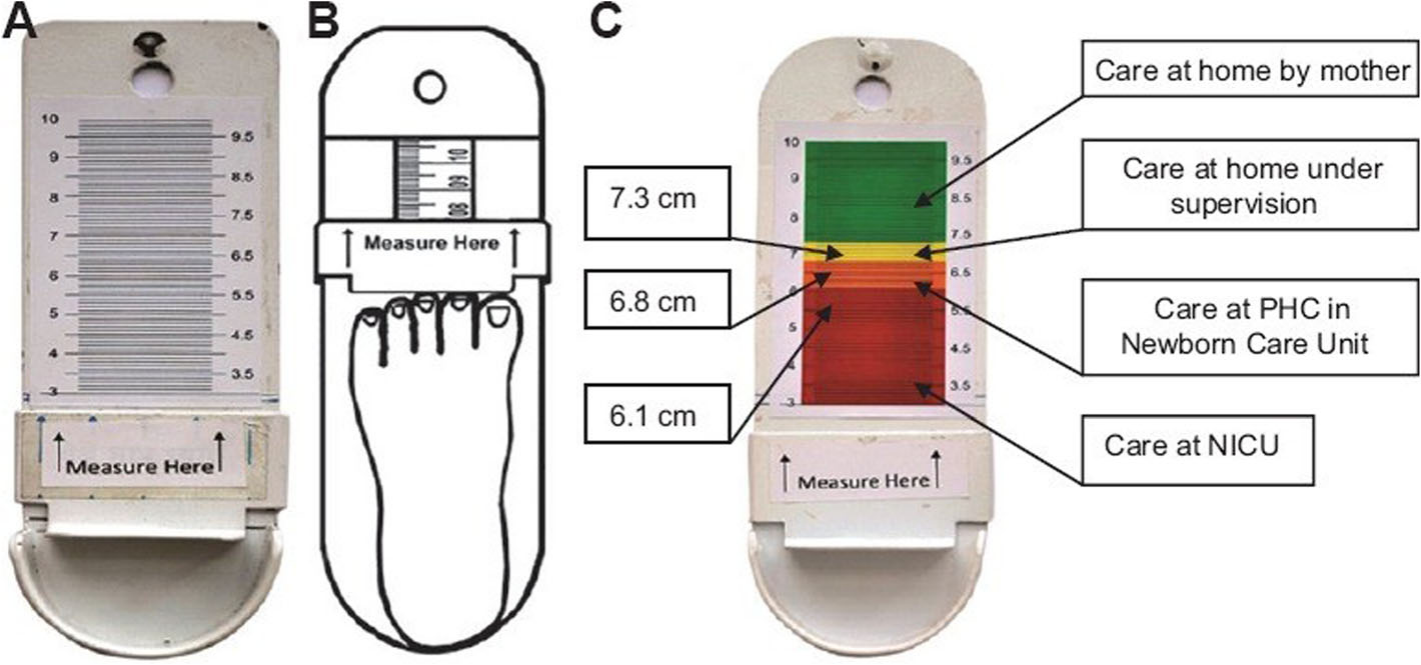
Measurement caliper (from Pratinidhi 2017 [11])

In a more recent study [11], conducted in a tertiary-level hospital in India, the investigators—having concerns about the accuracy of instruments such as plastic rulers—instead used a specially-made caliper (see Fig. 3). The intention was that such a measuring device and procedure could be used by community-based frontline health workers. Similar to the Nepal study cited above [2], Pratinidhi et al. [12] found that a cut-off of 6.8 cm performed best to differentiate by birthweight corresponding to survival risk. With this cut-off, 4% of those weighing < 2000 g (*n* = 23) were misclassified as normal weight and only 3.6% of those with birthweight ≥2500 g (*n* = 83) were misclassified as small (see Fig. 3).

The objective of our study was to determine if an intervention entailing provision to women late in pregnancy of a simple tool allowing them to classify their newborns as small increases uptake of key care practices (mediated through being exposed to recorded messages, heard on calling a toll-free number), notably: skin-to-skin thermal care and seeking treatment for possible danger signs. Implementation of the study as a cluster trial replicates conditions that would be present if this were delivered as a community-based program, in which one might expect amplification of effect through women sharing this information with friends and neighbors.

## Methods

The study was piggy-backed on a parent cluster-randomized controlled trial implemented in Sarlahi District (in the plains area of Nepal, close to the Indian border), investigating the efficacy of sunflower-seed oil massage on newborn survival [14]. Thirty-four participating government administrative areas (Village Development Committees (VDCs)) were randomized (independently from the parent trial), with 17 allocated to receive the foot-length card/ phone-message intervention and 17 to control. Randomization of clusters was done based on earlier data from the parent trial. Variables on which the randomization was restricted included:

- Size of birth cohort (i.e. number of live births),
- Low birth weight (i.e. proportion of babies weighing < 2500 g),
- Birth location (proportion born in facility),
- Skin-to-skin care (proportion of mothers reporting such care in the first week of life), and
- Care seeking (proportion of mothers reporting seeking care for infants in the first week of life).

Tolerance for determining “balance” of the five variables was set at 10%, i.e. a randomization was considered a candidate sequence if the ratio, r, of the variable of interest—when comparing the intervention (foot length card) to no intervention (absence of foot length card)—met the following criteria: 1/1.1 < r < 1.1. Among one million randomization sequences generated, 45,427 sequences met the above criteria, from which a single sequence was randomly selected.

All women in late pregnancy, aged 16 years and older, living in the 34 participating VDCs, and enrolled in the parent trial were eligible to participate in this study. Due to the design of the parent trial and the infrastructure in place for implementing it, field staff already had contact with women in late pregnancy, which enabled identification of eligible participants for this study.

A set of basic interventions common to all participating women, in both study arms, was provided during pregnancy, per government maternal-newborn health recommendations, including:

1. Promotional messages given using a visual aid on antenatal and essential newborn care. Content included maternal nutrition during pregnancy, danger signs and associated care-seeking, early and exclusive breastfeeding, clean and hygienic delivery including cord care, hand-washing, and thermal care of the newborn.
2. Clean delivery kit, consisting of a small bar of soap, a sterile blade and cord tie, a plastic disc on which to cut the cord, and a clean plastic sheet.
3. Iron folate supplements (90 days) and deworming medication (1 dose).
4. A small container of 4.0% chlorhexidine gel for daily application to their baby’s umbilical cord stump over the first days after birth.

The protocol also entailed monthly visits by study field-staff to the woman during pregnancy to record pregnancy status and to ask some basic questions about signs of morbidity during the previous 30-day period. At these visits, women also had their weight, blood pressure , and body temperature measured and recorded. Women with current signs or symptoms of morbidity were referred to the local health post or Primary Health Center. 

**Fig. 4:**
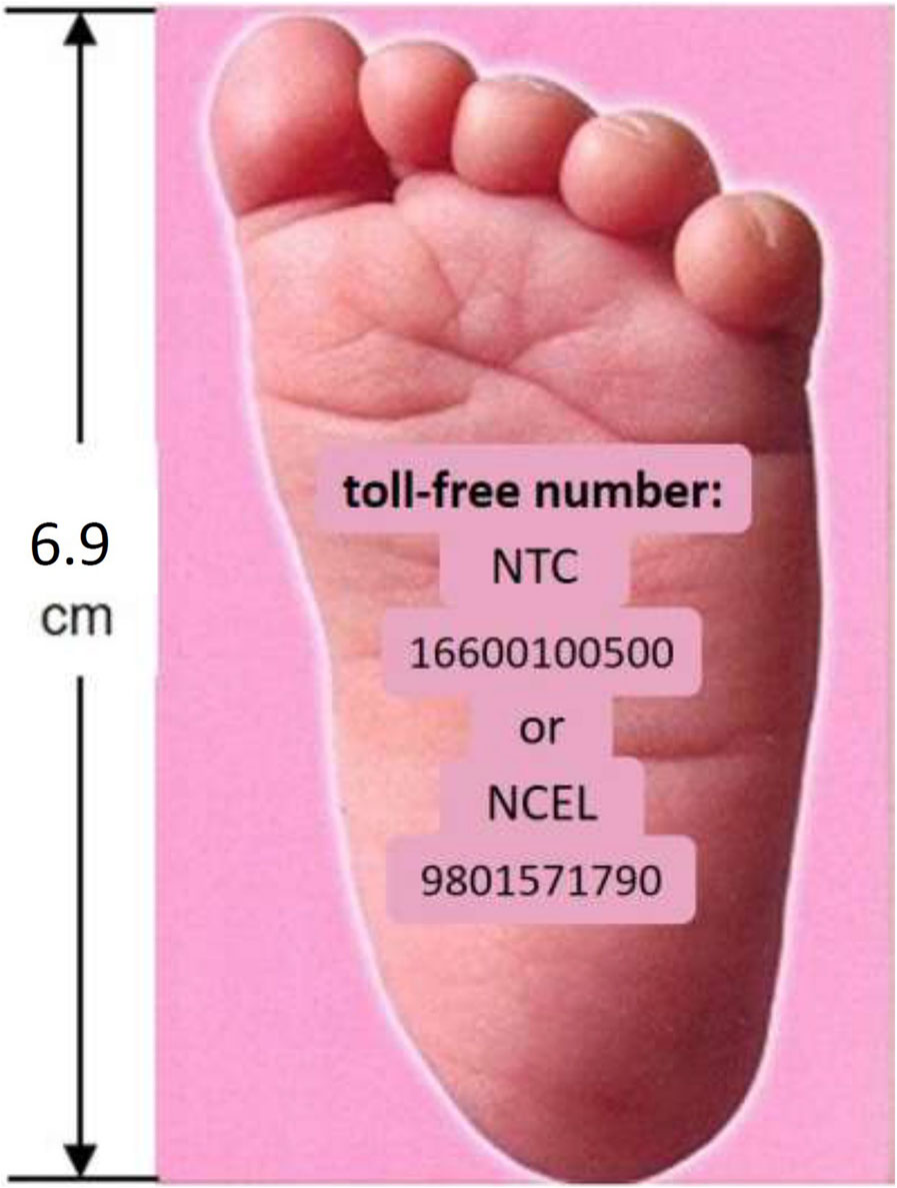
The Foot-Length Card

In the intervention arm, during scheduled late-pregnancy visits, study field staff gave the women the cards (see Fig. 4) with basic verbal instructions on their use in Maithili or Nepali , depending on their mother language (and gave the same information in written form). Mothers were told to lay their baby on a firm surface and place the card against the bottom of the baby’s foot to determine whether or not the foot was longer than the card and, if not, to call the toll-free number for helpful care instructions.^1^ On calling one of those numbers, the mothers or family members were able to listen to a 2-min recorded message, in Maithili or Nepali depending on which card they received (see full text in English in the online supplemental file).

The recording was intended to conveyed 3 cognitive and 2 affective messages, notably, cognitive: 1) avoiding allowing the baby to get *chilled* (best to maintain the baby skin-to-skin against the mother’s chest); 2) ensuring adequate feeding frequency (at least every 2.5 hours around the clock) and seeking assistance if the baby had difficulty feeding; and 3) recognizing potential danger signs and promptly *seeking medical care*; and affective: 1) urgency/ importance, and 2) self-efficacy (you can do this, and it will protect your baby).

The use of the card and the phone messages were pre-tested in another district with a group of pregnant and newly delivered mothers to determine acceptability of the procedure and comprehension of the messages. However pretesting did not include validation of accuracy of classification, using the foot-length card.

In both intervention and control clusters, study field-staff made repeated follow-up home visits: within 24 h of birth (for health facility births, the first postnatal home visit could be on day 3 or later) and then on days 3, 7 10, and 14. On each occasion, questions were asked about our principal endpoints (skin-to-skin care and treatment seeking).

In addition, during the first visit after birth (in most instances, within 24 h of birth), in intervention clusters field research staff inquired concerning card use. Among those reporting having used the card, field staff inquired concerning how the baby was classified (small/ not small), and whether or not care-givers called the toll-free number, and (for those reporting having made the call) they inquired concerning recall of the messages.

Study staff also used the card themselves, and classified the baby’s as having normal or short foot-length, and also during this visit they weighed the babies. In both intervention and control clusters, using instruments from the parent trial, mothers were queried about care-seeking behavioral outcomes (i.e. any skin-to-skin kangaroo mother care and any care-seeking from qualified health workers). From data gathered up to 14 days of life, it was determined whether or not either of the target care behaviors were reported.

Since the same study staff were involved in distributing the cards and in collecting information during postnatal visits, they were not blinded to intervention allocation status.

For sample-size determination, based on earlier data collected in this setting over the previous 4 years, we assumed that 30% of newborns would have birthweight < 2500 g, and that 30% of those in the non-intervention arm would report seeking care from a health worker and 25%, any skin-to-skin care. Using these inputs, to detect an absolute difference between intervention and control condition of 10 percentage points (which we judged to be a programmatically meaningful intervention effect size), with study power of 80%, a minimum of 1180 newborns would be required in each study arm.. We compared this required sample size (i.e. 1180 x 30% = ~ 354 LBW per group) under individual randomization to the expected yield given the already constrained and fixed parameters of the parent randomized trial. Specifically, under 1) VDC-randomization (fixed at a total of 34 units, or 17 per group), 2) an expected yield of ~ 35 LBW infants per cluster given available LBW rates and enrolment time remaining in the parent trial), and 3) an estimated coefficient of variation ranging from 0.1 to 0.2, we anticipated achieving approximately 75–90% power to detect the desired 10% difference in primary outcomes between the groups.

Baseline characteristics of the intervention and control groups were compared to examine the effectiveness of the randomization process and identify any potential confounding factors.

Analysis of study results consisted of two main components:

1. Assessing the impact of a communications intervention on two care behaviors with a potential to reduce risk for low birthweight newborns, and
2. Process documentation along the hypothesized causal pathway, expected to produce the intervention effect: *received card➔ measured baby’s foot➔ determined foot to be small➔ called number➔ recalled messages➔ adopted target behaviors*

For the assessment of behavioral impact, primary analysis was done by restricting the birthweight of newborns to < 2000 g, and with a less restrictive birthweight cut-off of < 2500 g. Analysis of effect size was a simple difference in proportions, which was assessed for significance using chi-square test, adjusting for cluster design, using the generalized linear model with binomial distribution and log link function.

For the process variables, analysis was done by disaggregating the variables in several ways, notably by birthweights of < 2000 g and < 2500, as determined by the field staff, and foot length assessed as ≤6.9 cm, as determined by the family and by the field staff. Use of the foot-length cards, calling the toll-free number, and recall of key messages were analyzed as simple proportions with 95% confidence intervals. All analysis was performed using Stata Version 14 (StataCorp, College Station, Texas).

## Results

**Fig. 5:**
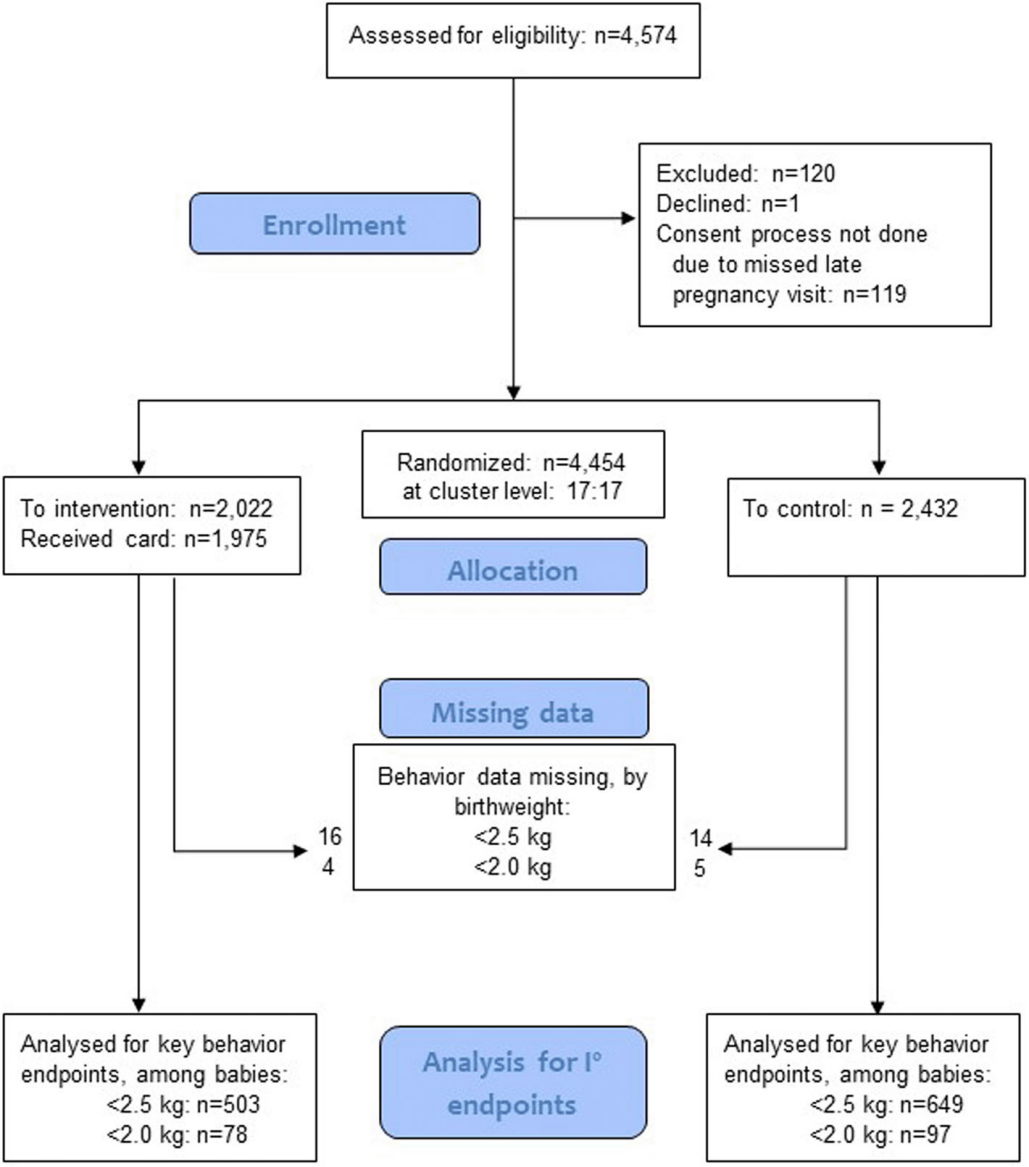
Participant flow diagram (34 clusters: 17 each randomized to intervention vs. control)

All 34 clusters were randomly assigned and included in the final analysis. As illustrated in the flow diagram (Fig. 5) a small number of potential individual participants were excluded or—due to missing data—were not included in the analysis of primary endpoints.

Study participants were recruited over the period July 2016 through January 2017, and each was followed up through two weeks post-delivery. As seen in Table 1, randomization of clusters achieved good balance on relevant socio-demographic and clinical characteristics.

**Table 1:** Baseline characteristics

		Comparison arm	Intervention arm
**Sex of the baby**	Male	53.2%	52.3%
	Female	46.8%	47.7%
	**Total**	**2432**	**2022**
**Age of mother at delivery (years)**	< 20	25.4%	25.0%
	20–24	43.5%	43.2%
	25–29	21.7%	22.5%
	30–34	6.9%	6.8%
	≥35	2.6%	2.5%
	**Total**	**2432**	**2142**
**≥1 antenatal visit**	No	13.3%	13.8%
	Yes	86.4%	86.1%
	**Total**	**2424**	**2141**
**Delivered in health facility**	No	47.4%	47.9%
	Yes	52.6%	52.1%
	**Total**	**2431**	**2142**
**Number of times pregnant, prior to this delivery**	None	26.5%	26.4%
	1	28.0%	28.4%
	2–4	39.6%	39.8%
	≥5	5.7%	5.3%
	**Total**	**2430**	**2141**
**Years of education – mother (years)**	None	63.4%	63.5%
	1–4	4.7%	4.3%
	5–9	19.1%	18.8%
	≥10	12.7%	13.4%
	**Total**	**2430**	**2141**
**Years of education – husband (years)**	None	40.1%	39.9%
	1–4	7.6%	5.8%
	5–9	30.8%	33.7%
	≥10	21.3%	20.5%
	**Total**	**2430**	**2139**
**Wealth terciles (by asset possession)**^**a**^	Low	23.1%	25.2%
	Medium	48.6%	48.1%
	High	28.2%	26.7%
	**Total**	**2432**	**2142**

^a^Calculated by combining 5 asset-related variables: mobile phone, television, land, latrine, and motorcycle. All variables dichotomized and then summed; scores then stratified into 3 categories; high, medium and low

As seen in Table 2, babies in the intervention arm who were born small (either < 2500 g or < 2000 g) were approximately 2.5 times more likely to receive skin-to-skin thermal care than those in the control arm. No significant difference was seen for care-seeking outside the home.

**Table 2:** Principal outcomes, care practices for low birthweight newborns (34 clusters)

	Skin-to-skin care				Sought outside care			
	Intervention % (n)	Control % (n)	RR (CI)	Intra-cluster correlation coefficients	Intervention % (n)	Control % (n)	RR (CI)	Intra-cluster correlation coefficients
**< 2500 g**	37% (503)	14.8% (649)	2.50 (2.01–3.10)	0.386	39.6% (503)	35% (649)	1.13 (0.97–1.31)	0.057
**< 2000 g**	33.3% (78)	13.4% (97)	2.48 (1.37–4.51)	0.337	42.3% (78)	38.3% (97)	1.05 (0.75–1.54)	0.157

The study also sought to open the black box on the expected causal mechanisms by which the intervention may produce its desired impact on care practices. As explained in the Methods section, our hypothesized causal process was as follows: *received card➔ measured baby’s foot➔ determined foot to be small➔ called number➔ understood and recalled messages➔ adopted target behaviors.* As is seen in Table 3, a high proportion of those in the intervention arm received the foot-length card and associated instructions, during a late pregnancy home visit. And, of those receiving the card, close to three quarters reported having used it to check their baby’s foot-length. Of those reporting not using the card, the reasons given included: 1) lost or forgot to use the card – 68%, 2) gave birth at the hospital – 18%, 3) other responses – 14%.

**Table 3:** Process analysis (restricted to the intervention arm)

	n	% (95%CI)	Total N
Received card and instructions	1953	96.6 (95.8–97.4)	2022
Used card to check baby’s foot-length	1443	73.9 (71.9–75.8))	1953
**Judged baby’s foot smaller than the card**	**24**	**1.7 (1.0–2.3)**	**1443**
Called toll-free number & listened to messages	62	3.2 (2.4–3.9)	1953
Called number & listened to messages, among < 2500 g	22	4.6 (2.7–6.5)	476

**Fig. 6:**
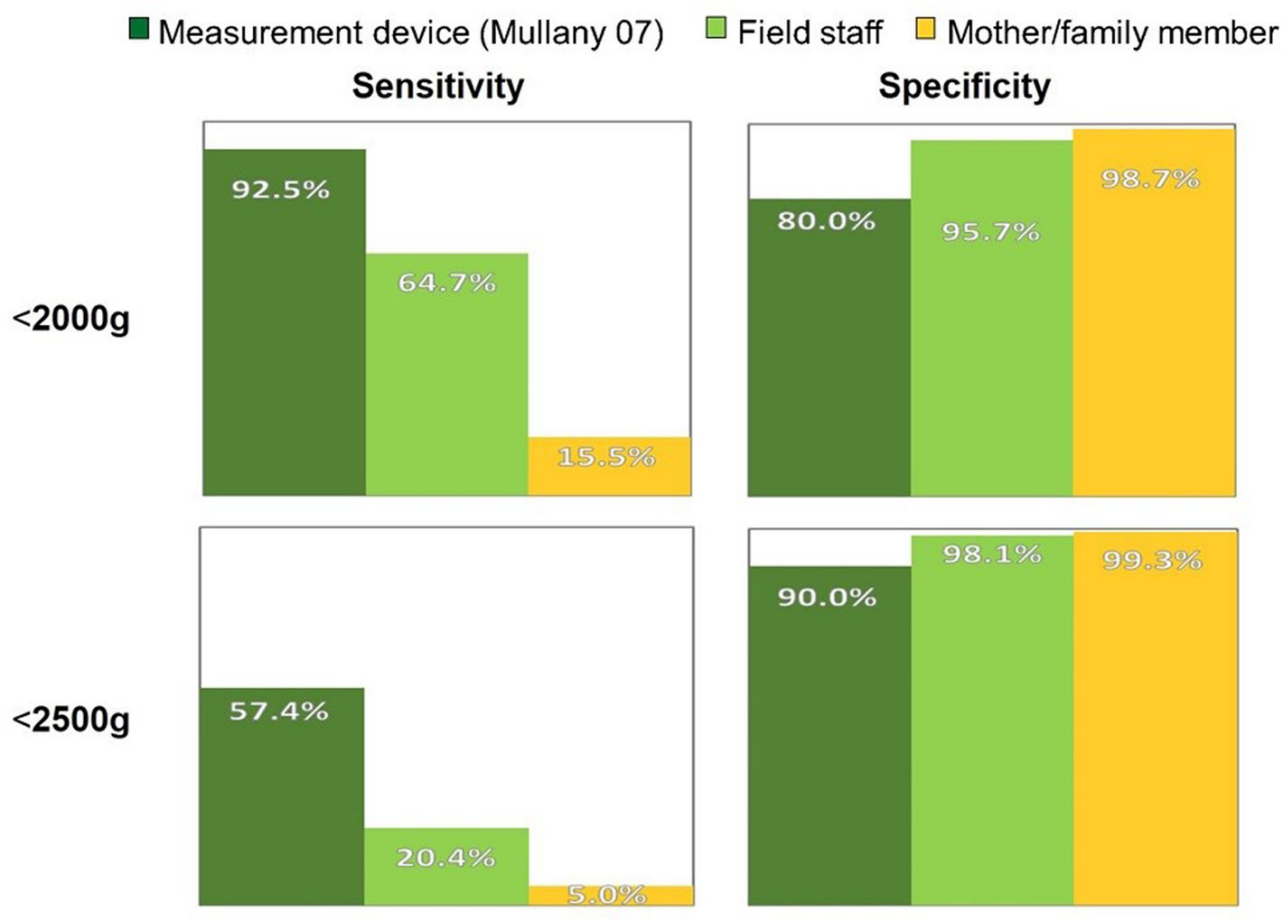
Classification Accuracy for Low Birthweight (using a 6.9 cm cut-off)]

Among those checking foot-length, only a very small proportion (1.7%, 95% CI: 1.0–2.3) reported that they found the baby’s foot to be shorter than the card. This was in marked contrast to findings of earlier anthropometric assessments in this population, and is discordant with results from field-staff use of the cards (see Fig. 6). Among the very small number of mothers reporting having called the toll-free number, message recall was relatively good (results not presented here), however given that such calls were made for only a very small proportion of low birthweight babies, the intervention—as designed—could not be expected to produce a discernable population-level effect, regardless how well understood and recalled the messages were.

As illustrated in Fig. 6, from data in an anthropometric study conducted in this population [3], using their measurement device and a foot-length cut-off of 6.9 cm, for infants born < 2000, this criterion had a sensitivity of 87.5% (i.e. it would misclassify only 12.5% of these very small newborns as normal weight). For a weight threshold of 2500 g, the same cut-off had a specificity of 94.2% (i.e. of those with foot-length ≥ 2500, only 5.8% would be misclassified as small). By contrast, in our study when mothers or other family members used the foot-length card to classify their newborns, they missed 84.5% of those that were actually < 2000 g (classifying them as normal). When field staff used the card, their performance was intermediate between use of the measuring device and mother or family member classification, but they still misclassified 35.3% of those < 2000 g as normal weight.

## Discussion

The main outcome measures for the trial were reported skin-to-skin thermal care and seeking care for the newborn from a service provider outside the home, restricted to low birthweight newborns (using two thresholds: 2000 g and 2500 g). For care-seeking, the study findings do not support there being any intervention effect. For reported skin-to-skin care, however, the trial found a relative risk of 2.50 (95%CI: 2.01–3.1), with the 2500 g threshold, and 2.48 (95%CI: 1.37–4.51) for 2000 g. One could—naively—infer that the foot-length card screening/ recorded-message intervention was effective in improving thermal care practices, notably skin-to-skin care. However, the study also documented intermediate steps in the presumed causal chain through which the effects of the intervention could be mediated. The weak link in that chain turned out to be classification accuracy using the card. Even when used by research field staff, who had the opportunity to build their skills assessing multiple newborns, use of the card poorly approximated the results obtained with the specially designed measurement device used in the earlier anthropometric study [2], seen in Fig. 1. In the hands of mothers and other family members, use of the card to assess foot-length—as a basis for classifying their newborns as at risk due to low birthweight—performed very poorly, misclassifying 84.5% of very small newborns (< 2000 g), and fully 95% of those < 2500 g, as normal weight. So any enhanced adoption, for these small babies, of better thermal care practices cannot be attributed to parents identifying their baby as small using the card, calling the number, hearing the messages, understanding, and applying them.

The study used a randomized control trial methodology, sample size was adequate for the outcomes assessed, and good balance was achieved between treatment and control arms. One constraint was that it was important to interfere as little as possible with the parent study on which our trial was superimposed, minimizing additional engagement with study participants or measurement. This limited us to potentially relevant outcomes that were already being measured in the parent trial. Had the study been conducted as a stand-alone trial, a fuller range of outcome measures better approximating the objective of this intervention strategy could have been used.

Formative work had been done in a rural Nepal setting to validate that the phone script used was well understood by pregnant women and women who had recently given birth. However, in retrospect, an unwarranted assumption was made that measurement and classification by mothers or other family members, using a card measuring 6.9 cm, would closely approximate results obtained by field research staff with a purpose-built device as used in the earlier anthropometric study. This was not tested in advance of the trial.

The findings appear paradoxical, on the face of it. On the one hand, among small newborns in the intervention arm, skin-to-skin thermal care was practiced considerably more than in the control arm. But, based on the process measures, it is clear that this cannot be attributed to correct classification, calling the number, hearing the messages and applying them. So what can account for the apparent impact of the intervention?

At the time of the first postnatal visit, of those who had received the card, only 3.2% (62/ 1953) reported having called the toll-free number and heard the recorded messages. It is possible that over the following days, more of parents called the number, regardless of what they found using the foot-length card, and this—conceivably—could have influenced thermal care practices. However, any calls that may have occurred subsequent to the first postnatal home visit were not assessed in this study so we are not in a position to further explore this hypothesis. An alternative explanation is that some aspect of the interaction between the field research staff and study participants, either during the late pregnancy visit or at the time of the initial contact after birth, could have sensitized mothers somehow to the importance of skin-to-skin care for very small newborns, regardless how they classified them using the card. Study co-investigators who were involved in field supervision have reviewed the procedures used but have failed to identify a plausible explanation for how this could have occurred, based on how field contacts were implemented. Of course, even with confidence intervals around the relative risk point estimate that do not overlap 1.0, it certainly remains possible that such a result could have arisen through pure chance. In our judgement, this is the most credible explanation for the study finding of an apparent intervention effect.

As noted in the Introduction, newborns of low birthweight account for the majority of neonatal deaths [3], many of which could be prevented with good attention to thermal care [4], optimal newborn breastfeeding practices [5], and prompt medical attention for danger signs. In settings where many births still occur at home, not attended by professional health workers, there is a compelling program logic to trying to find ways of identifying these small newborns and ensuring the needed care practices. In such a context, simple tools that could allow visiting community health workers, or mothers themselves, to identify higher risk babies, have real appeal. Salter spring scales, made specifically for weighing infants, are inexpensive and comparatively simple to use. However, for accurate results they do require skills and proper procedure; earlier efforts in Nepal to have FCHVs make postnatal home visits, which included weighing newborns, faltered, both due to low early home visitation rates and difficulties the FCHVs had using the scales [12].

Newborn anthropometric studies have been done in a number of settings, assessing correlation between birthweight and a variety of other measures, including foot-length, and have generally shown reasonably good test characteristics for foot-length [2, 11, 15–18]. Although most of these studies did not find foot-length to be the best proxy for birthweight, foot-length measurement has seemed more practical for field use than other measures that generally performed better (e.g. chest or head circumference).

Two groups have tested strategies involving having CHWs assess foot-length, during home visits. As noted in the Introduction, in a field study in Tanzania [15], during postnatal home visits, CHWs assessed foot-length to classify babies and, if small, gave counseling messages on needed care. In that study, CHWs first classified cases using the card, then measured the baby’s foot using a hard ruler. Subsequently, the field researcher independently assessed foot-length using the card, and then measured using the ruler. Inter-rater reliability was assessed, comparing length measures made by the CHWs and the field researcher. Moderate reliability was found, with a kappa of 0.53. On average, CHWs assessed foot length at 0.3 cm shorter than measures by the field researcher, thus overestimating the number of small babies (opposite to the problem found in the current study). In this Tanzanian study, length measures were not assessed against weight (a more appropriate gold standard).

Another study, conducted in Bangladesh [19], assessed performance of CHW assessments done during early postnatal home visits, which included foot length measures with a hard ruler, using the same procedures as in the Tanzanian study. For the Bangladesh study, however, the primary interest was correlation with gestational age, as determined by early pregnancy ultrasound. Foot-length, as measured by CHWs, performed poorly in this regard.

With such findings, along with the results of the current study, appealing though the idea may be, having family members or CHWs assess risk associated with low birth weight using foot-length as a proxy does not appear to be a promising strategy. The challenge remains of how best to identify babies at risk, and to mitigate such risks through targeted efforts to ensure optimal care practices. In principle, for institutional births, mothers of all babies born at a weight that puts them at higher risk need to be given good counseling, before discharge home, on key practices, notably: thermal care, breastfeeding, and danger sign recognition and associated prompt care-seeking. For populations in which home births, not attended by professional health workers, remain common, other strategies are needed. Counseling during late pregnancy antenatal contacts should certainly include essential newborn care messages, emphasizing particular risks faced by smaller-than-average newborns.

## Conclusion

On one of the two primary outcomes in this community-based cluster randomized trial, skin-to-skin thermal care, participants in the study arm were 2½ times more likely to report the practice than those in the control arm. However, process measures along the hypothesized causal chain document that the intervention did not perform well: classification of newborns as small using a card 6.9 cm long (a cut-off determined from an earlier anthropometric study in this setting) performed poorly as a proxy for birth weight. As a result only a very small proportion of those who could have benefited from doing so followed up to make a call to receive information on care of these higher-risk newborns (including on skin-to-skin care). So, very few of those with small newborns in the intervention arm were actually exposed to the health messages that constituted the core of the intervention. We cannot rule out the possibility that some aspect of conduct of the study within the intervention arm could have influenced women in the intervention arm to adopt this thermal care practice. But—on review of study field procedures—it seems more plausible to us that this result is attributable to chance.

Making an unbiased, reasonably accurate classification of foot-length using a card, as we attempted to have household members do in our study, is not as straightforward as we expected. There is still a need, in Nepal and other similar settings, to reach women giving birth at home, to inform and empower them to take action to reduce risk for babies born very small, but a different approach will be needed.

## Supplementary information

**Additional file 1.** Annex: English translation of voice recording with care messages for small newborns. (DOCX 14 kb)

## Data Availability

The datasets used and/or analysed during the current study are available from the corresponding author on reasonable request.

## Abbreviations

CHW: Community Health Worker
FCHV: Female Community Health Volunteer
VDC: Village Development Committee

## Footnotes

1 [note refers to figure 4] Text of instructions given (in English): Concerning this card … For babies born very small there can be more danger than for normal sized babies. This card is used to help to tell if your baby is very small. This is how to use it … After your baby is born (whether at home or health facility), you should check to see if your baby’s foot is longer than the card. Placing the baby on a flat surface, put the card against the sole of the baby’s foot, resting the bottom of the card on the surface the baby is lying on. Then you can check whether or not the baby’s toes extend beyond the end of the card. If the baby’s foot is NOT longer than the card or even if the foot is longer but the baby still seems to you to be very small, call one of the numbers on the card. The call is free. When you call, you will be able to listen to a message on special care needed for the baby to help protect him or her. You can listen as many times as you like.
