# Supplementary material for: Community-based cluster randomized controlled trial: empowering households to identify and provide appropriate care for low-birthweight newborns in Nepal
Source: BMC Public Health. 2020 Aug 24;20:1274. doi: 10.1186/s12889-020-09317-w (PMC7446145; doi:10.1186/s12889-020-09317-w)
Supplement: Supplementary file 1 — Additional file 1. Annex: English translation of voice recording with care messages for small newborns. (DOCX 14 kb) [file 12889_2020_9317_MOESM1_ESM.docx]

**Annex: English translation of voice recording with care messages for small newborns.**

It’s very good that you have called.

Is your newborn's foot smaller than the foot-length card or does he/ she seem to be very small? If so, your baby may have been born earlier than the proper time or may have low birthweight and her/ his health could be in danger. Now you should do three things to reduce risk to your newborn.

**First**: Breast-feed your baby day and night at 2 hourly intervals. A small baby may have difficulty sucking breast milk. If so, then express breast milk into a bowl and feed the baby, using a spoon.

**Second**: For very small newborns, it is very important to keep them warm. If your newborn is exposed to cold, her/his life can be endangered due to chilling. Therefore, the mother should place the baby between her breasts and cover her/ him with a warm cloth to keep the baby warm. To give the mother a break, other family members can keep the baby on their chests, covered, from time to time. Keeping the baby skin-to-skin like this will decrease risks to the baby and will also help the baby to gain weight more quickly and become strong and healthy.

Check the baby’s condition frequently.

**Third**: If your baby can’t suck well, doesn’t move as much as you would expect, or has difficulty breathing, she/ he may be sick. If you see such symptoms, immediately take your baby to the health facility or health worker. During travel to the health facility, keep your newborn skin-to-skin, on your chest.

Note, once again, if your newborn's foot is smaller than the foot-length card or if she/ he seems very small, your baby is at risk.

- She/ he should be fed every two hours, day and night.
- Protect your baby from chilling by keeping him/her skin-to-skin, between the mother’s breasts.
- If there is difficulty in feeding (even with a spoon) or breathing, or your baby seems less active than before—immediately take her/him to the health facility or to a health worker.

Your family members also should listen to these messages.

If you want to listen again, please press 1 on the mobile keypad

Thank you
